# Effects of Controlled Atmosphere Conditions on the Quality Characteristics, Physicochemical and Antioxidant Properties of Pork Bone Broth

**DOI:** 10.3390/foods15071188

**Published:** 2026-04-01

**Authors:** Ying Su, Junli Dong, Qian Deng, Long Zhang, Jing Li, Jie Chen

**Affiliations:** 1 School of Food Science and Technology, Jiangnan University, Wuxi 214122, China; 2 State Key Laboratory of Food Science and Resources, Jiangnan University, Wuxi 214122, China; 3 Guangdong Midea Consumer Electric MFG. Co., Ltd., Foshan 528000, China

**Keywords:** sensory quality, nutritional composition, flavor profile, emulsion stability

## Abstract

Controlled atmosphere (CA) is widely employed to preserve perishable foods, yet its potential effects on the quality of thermally processed bone broth remain poorly understood. This work systematically investigated the influences of ventilation time (0, 1, and 3 s), ventilation frequency (30, 60, 90, and 110 cycles), and cooking duration (25, 30, 38, and 45 min) on the overall quality of pork bone broth. A single-factor experimental design was adopted with three replications per treatment. Results showed that CA treatment effectively improved the sensory properties of pork bone broth, including color, aroma, and taste. Different CA processing parameters differentially affected the accumulation of diglycerides, proteins, peptides, amino acids and lipid oxidation-related flavor compounds, as well as antioxidant activities and emulsion stability. Specifically, prolonged ventilation promoted the accumulation of diglycerides and medium-sized peptides (1–7 kDa) but concurrently reduced solids, fat content, and ABTS radical scavenging activity, suggesting a trade-off between flavor precursor generation and oxidative stability. Furthermore, most quality indicators initially increased with rising ventilation frequency but subsequently declined at excessive levels, with optimal values attained at moderate frequencies. Notably, CA conditions that enhanced the formation of desirable flavor compounds also increased the accumulation of lipid oxidation byproducts, highlighting a critical balance required for achieving optimal product quality. Ultimately, it was found that a ventilation time of 1 s, a ventilation frequency of 60 cycles per minute, and a cooking duration of 30 min maximized the benefits of controlled atmosphere (CA) processing, thereby achieving optimal sensory properties, flavor profiles and nutritional composition in pork bone broth. This study provides fundamental data to support the development and quality regulation of thermally processed meat broths.

## 1. Introduction

Global pork production is a pivotal sector of the agricultural economy, with output of 120 million tons in 2021, accounting for 34% of the world’s total meat production [1]. China alone contributed 45% of this supply [1]. Such extensive production yields approximately 130 million tons of bone by-products annually [2]. Nevertheless, the advanced processing rate for pork, cattle, and poultry bones remains critically low, at less than 1% [3,4]. Comprising about 12.9% of the carcass weight, pork bones are not only abundant in conventional nutrients such as proteins and lipids but also exceptionally rich in collagen, choline, and essential minerals, including calcium and phosphorus [5]. This nutrient-dense composition provides considerable potential for the development of high-value products like bone powder, bone paste, and bone protein hydrolysates, which are increasingly valued for their health-promoting attributes [6,7,8,9]. Despite growing interest in nutrient-rich pork bone broth, advanced processing technologies tailored to this specific product remain underdeveloped [3].

Pork bone broth is a traditional culinary product valued across various cultures for its rich flavor, pronounced umami character, and wide array of nutritional benefits [10].

It was found that the structural integrity of muscle fibers in vacuum-cooked pork was severely compromised, resulting in marked deterioration of the myofibrillar structure, which directly led to a dry, tough texture of the cooked product [11]. However, this observed trend indirectly indicates that vacuum cooking treatment facilitates the leaching and dissolution of water-soluble components from the bone matrix. Wang Yao et al. [12] demonstrated that pork bone broth presents distinct taste and aroma profiles when prepared under varying cooking temperatures and controlled air circulation regimes; specifically, broth cooked at 96 °C with continuous air circulation exhibited the most intense meaty aroma and optimal overall sensory acceptability. Therefore, the final quality of the broth is highly dependent on cooking parameters—including time, temperature, and notably, the oxidative environment—that collectively influence nutrient extraction efficiency, flavor development, and emulsion stability [5]. For instance, Katsuno et al. [13] found that dissolved oxygen in the system interacts with aminoreductone (Maillard reaction product) formed during the thermal processing of milk—leading to its oxidative degradation and the reduction in its concentration in the milk matrix. Besides, controlled ventilation during bone broth cooking dynamically modulates both the bulk temperature of the broth matrix and its dissolved oxygen concentration, thereby directly altering the rate and extent of lipid oxidation within the system. Liquid level fluctuations induced by controlled ventilation attenuate the progression of the high-temperature Maillard reaction in the broth system. In parallel, a pressurized cooking environment induces a marked reduction in particle size and a narrower size distribution of fat globules within the protein–lipid emulsion matrix of bone broth, mitigating the coalescence of these fat globules and concurrently enhancing the leaching and thermal degradation of water-soluble proteins in the broth. The enhanced surface activity of these degraded protein fractions enables tighter anchoring to the lipid phase at the oil–water interface, which facilitates the formation of a highly stable protein–lipid composite emulsion [14]. Controlled atmosphere (CA) technology, defined as the intentional manipulation of gas composition within an enclosed environment, is widely employed in food storage to extend the shelf life of perishable items by mitigating oxidative degradation and inhibiting microbial growth [15,16,17,18]. Despite its established role in food preservation and selected processing applications [19,20], the potential of CA technology to actively improve cooking processes—particularly in complex colloidal systems such as bone broths—has been rarely explored, representing a notable research gap.

While prior research has predominantly emphasized ingredient selection and conventional cooking methods for improving the nutritional and sensory qualities of bone broth [21,22], the strategic integration of CA conditions during cooking offers a novel avenue for optimization. Traditional high-temperature cooking, though effective for nutrient extraction, often facilitates degradation of heat-sensitive aromatic compounds and accelerates undesirable lipid oxidation [23]. In contrast, cooking within a sealed, oxygen-limited environment may suppress heat-induced oxidation but could also restrain the formation of desirable flavor compounds typically generated through MR and other oxidative pathways [24,25]. This underscores a critical technological trade-off. The previous work by Su et al. [14] has identified and elucidated clear distinctions between CA and non-controlled atmosphere (NCA) processing. Thus, a systematic investigation is imperative to elucidate how CA conditions can be optimized to steer these biochemical reactions toward favorable outcomes in bone broth processing.

It has been hypothesized that modulation of key CA parameters—including ventilation time, ventilation frequency, and cooking duration—can systematically regulate the kinetics of lipid oxidation, the Maillard reaction, protein degradation, and emulsion stability, thereby significantly enhancing the sensory attributes, nutritional composition, flavor profiles, and antioxidant capacity of pork bone broth relative to non-controlled atmosphere (NCA) treatments. This study aims to address this research gap by systematically investigating the influence of key CA parameters—ventilation time (0, 1, 3 s), ventilation frequency (30, 60, 90, 110 cycles), and cooking duration (25, 30, 38, 45 min)—on the sensory attributes, nutritional composition, flavor profile, microstructure, emulsion stability, and antioxidant capacity of pork bone broth.

## 2. Materials and Methods

### 2.1. Materials and Reagents

Pork keel bones and fresh ginger were sourced from Datonghua Shopping Center Co., Ltd. (Wuxi, China). All pork bones were from the same batch of healthy commercial adult pigs. Cooking wine was obtained from Jiangsu Hengshun Vinech Industry Co., Ltd. (Zhenjiang, China). Edible salt was supplied by China National Salt Industry Group Co., Ltd. (Beijing, China), and drinking water was provided by Jiangsu Dongting Mountain Mineral Water Group (Wuxi, China). All general chemical reagents, including boric acid, sodium hydroxide, methyl red, bromocresol green, hydrochloric acid, anhydrous sodium carbonate, copper sulfate pentahydrate, potassium sulfate, concentrated sulfuric acid, petroleum ether, undecanoic acid, sodium chloride, potassium persulfate, ferric chloride, and sodium nitrate, were procured from Sinopharm Chemical Reagent Co., Ltd. (Shanghai, China). Trichloroacetic acid was acquired from Damas Beta (Shanghai, China). Organic solvents—ethanol, chloroform, n-hexane, ether, methanol, and acetonitrile—were supplied by Titan Scientific Co., Ltd. (Shanghai, China). ELISA kits for quantification of diglycerides (DG) and triglycerides (TG) in pork were purchased from Shanghai Yuanxin Biotechnology Co., Ltd. (Shanghai, China). Additionally, the following specialty biochemical reagents and standards were obtained from Yuanye Bio-Technology Co., Ltd. (Shanghai, China): 2,2′-azinobis-(3-ethylbenzthiazoline-6-sulfonate) (ABTS), 6-hydroxy-2,5,7,8-tetramethylchroman-2-carboxylic acid (Trolox), 2,4,6-tris(2-pyridyl)-1,3,5-triazine (TPTZ), as well as peptide standards comprising glycine–glycine–glycine (Mw: 189 Da), glycine–glycine–tyrosine–arginine (Mw: 451 Da), bacitracin (Mw: 1450 Da), aprotinin (Mw: 6511 Da), and cytochrome C (Mw: 12,384 Da).

### 2.2. Preparation of Pork Bone Broth

**Figure 1:**
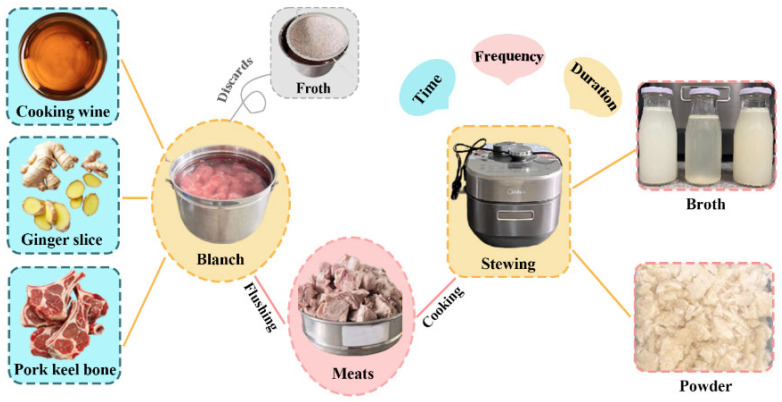
Preparation process of pork bone broth.

The detailed procedure for preparing pork bone broth is illustrated in Figure 1. The methodology was slightly modified based on the work of Hou Miaomiao et al. [26]. Briefly, 2000 g of pork keel bones were thoroughly combined with 4000 g of potable water, 5% (*v*/*v*) cooking wine, and 3% (*w*/*w*) fresh ginger slices in a stainless-steel pot. The mixture was heated using a C22-MICCA901 induction cooker (Midea, Foshan, China) (The pressure-relief mechanism of the pressure cooker lid was modified to introduce air into the vessel interior, thereby establishing a controlled atmosphere. The airflow stability, temperature stability, pressure stability, and operational safety were evaluated subsequent to the modification; all test results complied with relevant standards.) at 2100 W for 15 min to achieve initial boiling and remove impurities. Subsequently, the bones were carefully rinsed under cold running water, drained, and transferred to an MY-S572N pressure cooker (Midea, Foshan, China). Additional potable water was added at a fixed mass ratio of 1:2 (bones to water). As outlined in Table 1, the cooking process was systematically designed to evaluate the effects of key parameters under controlled conditions at 110 °C, including ventilation time (0, 1, and 3 s), ventilation frequency (30, 60, 90, and 110 cycles), and cooking duration (25, 30, 38, and 45 min). The resulting samples were labeled based on the experimental variables: T0, T1, and T3 (representing ventilation time); F30, F60, F90, and F110 (representing ventilation frequencies); and D25, D30, D38, and D45 (representing cooking duration). After pressure cooking, the broths were immediately filtered through a 60-mesh sieve to remove residual particulates and impurities. The filtrates were aseptically packaged into food-grade polypropylene bottles and subjected to freeze-drying for 48 h using a Scientz-50F/A freeze dryer (Ningbo Scienrz, Ningbo, China). The resulting lyophilized powders were stored in a desiccator at room temperature to remain stability until further analysis. All samples were prepared in triplicate to ensure the reliability of the results.

### 2.3. Sensory Evaluation

Sensory evaluation was performed according to the method reported by Begum et al. [27], with slight modifications. The training methods of sensory evaluators refer to Ye Haixia et al. [28]. Sensory assessment was conducted by a trained panel from Jiangnan University (Wuxi, China). Twenty healthy panelists with prior professional sensory evaluation experience were recruited (10 males and 10 females, aged 20–55 years). Prior to sensory analysis, the broth was kept at a constant temperature of 60 °C. Subsequently, 0.6% (*w*/*w*) sodium chloride was added and fully dissolved. The resulting mixture was then transferred into transparent sample cups. All samples were labeled with random three-digit codes and presented to the panelists in random order under blind conditions to minimize subjective bias. All sensory analyses were performed in triplicate. Panelists evaluated key attributes, including color, odor, taste, mouthfeel, and overall liking, using a structured 10-point hedonic scale. All participants provided written informed consent in accordance with the Chinese National Standard GB/T 10220-2012 [29]. The study protocol received ethical approval from the Ethics Committee of Jiangnan University (Reference No.: JNU202412RB001).

### 2.4. Basic Component Content

#### 2.4.1. Solid Content

The solids content has been adjusted slightly by Guo et al. [24]. To avoid potential nutrient degradation caused by high-temperature treatments and to align with subsequent nutritional analyses, this study utilized freeze-drying instead of conventional oven-drying for the determination of solid content. Freshly prepared broth was filtered and uniformly aliquoted into pre-weighed 150 mm freeze-drying dishes (weight denoted as M_dish_). The weight of the broth sample (M_broth_) was accurately recorded before subjecting it to lyophilization. After lyophilization, the total weight of the dried residue and dish (M_dry_) was measured. The total solids content was calculated according to Equation (1):

(1) $$\mathrm{Total}\;\mathrm{solids}\;\mathrm{content}\;(\%)\;=\;\frac{M_{\mathrm{dry}}\;-{\;M}_{\mathrm{dish}}}{M_{\mathrm{broth}}}\;\times \;100$$

#### 2.4.2. Protein Content

The nitrogen content of the broth samples was determined using a K9840 Kjeldahl nitrogen analyzer (Hanon Scientific Instruments Co., Ltd., Dezhou, China). Total protein content was subsequently calculated based on the nitrogen results, in compliance with the National Food Safety Standard of China (GB 5009.5-2016) [30,31].

#### 2.4.3. Fat Content

Fat content was determined employing a SZF-06A Soxhlet extraction system (Xinrui Instruments Co., Ltd., Shanghai, China). Freeze-dried broth powder was subjected to continuous extraction with petroleum ether, following the official method prescribed in GB 5009.6-2016 [32,33].

#### 2.4.4. Diglycerides and Triglycerides Content

The concentrations of diglycerides (DG) and triglycerides (TG) were quantified using species-specific ELISA kits for porcine DG and TG. Each sample was analyzed in duplicate. Briefly, 50 µL of standard solutions at varying concentrations were added to standard wells, while 10 µL of sample combined with diluent were transferred to sample wells. Then, 100 µL of horseradish peroxidase (HRP)-conjugated detection antibody was added to all wells except blanks, and the plate was incubated at 37 °C for 1 h. After thorough washing, 50 µL each of substrate solution A and B were added to each well, followed by incubation in the dark at 37 °C for 15 min. The enzymatic reaction was terminated by adding 50 µL of stop solution, and the absorbance was immediately measured at 450 nm using a microplate reader (SpectraMax 190, Molecular Devices, CA, USA).

### 2.5. Fatty Acid Content

The fatty acid composition was performed according to the method of Wang Zhenwei et al. [34]. In total, 200 mg of freeze-dried broth powder was accurately weighed and combined with 2 mL of internal standard solution (5 mg/mL undecanoic acid in methanol) and 10 mL of lipid extraction solvent (chloroform: methanol = 4:1, *v*/*v*). The mixture was subjected to overnight extraction at room temperature with continuous shaking. After centrifugation, the supernatant was collected, and the residue was re-extracted with an additional 10 mL of extraction solvent. The combined extracts were evaporated to dryness in a water bath maintained at 65 °C. For derivatization, 4 mL of 2% (*m*/*v*) sodium methoxide solution was added to the dried lipid extract, and the mixture was incubated at 60 °C for 30 min to facilitate transesterification. Then, 4 mL of 25% boron trichloride in ether was added, and esterification was carried out at 60 °C for another 30 min. After cooling, 5 mL of saturated sodium chloride solution and 2 mL of n-hexane were added to partition the fatty acid methyl esters into the organic phase. The upper n-hexane layer was collected, filtered through a 0.22 μm membrane, and subjected to gas chromatography analysis.

### 2.6. Amino Acid Composition

The method of determining amino acid composition refers to Feng et al. [35]. Sample pretreatment for free amino acids (FAA): A 4 mL aliquot of broth was mixed thoroughly with 4 mL of 10% (*m*/*v*) trichloroacetic acid solution and allowed to stand for 30 min to precipitate proteins. The mixture was then centrifuged at 10,000 rpm for 10 min using a high-speed centrifuge (GL-10MD, Hunan Xiangyi Centrifuge Instrument Co., Ltd., ChangshaChina). Subsequently, 400 μL of the supernatant was carefully collected for subsequent amino acid analysis.

Sample pretreatment for total amino acids (TAA): Another 4 mL portion of broth was placed into a sealed hydrolysis tube, mixed with 8 mL of 6 mol/L hydrochloric acid, and purged with nitrogen gas for 30 min to eliminate oxygen. The tube was sealed under nitrogen atmosphere and hydrolyzed at 120 °C for 22–24 h. After cooling, the hydrolysate was neutralized, diluted to a final volume of 25 mL with deionized water, and filtered. The resulting solution was centrifuged, and 400 μL of the supernatant was collected for analysis.

High-performance liquid chromatography (HPLC) analysis: Amino acid quantification was performed using an Agilent HP1100 high-performance liquid chromatography system (Agilent Technologies, Santa Clara, CA, USA) equipped with a Venusil-AA column (100 A, 4.6 mm × 250 mm, 5 μm). The mobile phase consisted of (A) sodium nitrate–acetonitrile buffer (pH 6.5) and (B) 80% acetonitrile. The flow rate was maintained at 1.0 mL/min, the column temperature was set at 40 °C, and detection was carried out at 254 nm.

### 2.7. Peptide Molecular Weight Distribution

The molecular weight distribution of peptides was performed according to the method of Ma et al. [36]. 10 mg of the freeze-dried broth powder was dissolved in 1 mL of ultrapure water and thoroughly vortexed. The solution was then centrifuged at 10,000 rpm for 10 min to remove insoluble material, and the supernatant was filtered through a 0.22 μm membrane. The filtrate was analyzed using a HPLC system (Waters 2695, Waters Corp., Milford, MA, USA) coupled with a DAWN HELEOS 8+ multi-angle light scattering (MALS) detector (Wyatt Technology Co., Ltd., Santa Barbara, CA, USA). Separation was performed on a TSK-Gel G2000SWXL size exclusion column (7.8 mm × 300 mm, Tosoh Corporation, Tokyo, Japan) maintained at 30 °C. The mobile phase consisted of 45% (*v*/*v*) acetonitrile containing 0.1% (*v*/*v*) trifluoroacetic acid, delivered at a flow rate of 0.5 mL/min. Detection was carried out at 214 nm. A calibration curve was constructed using standard peptides covering a broad molecular weight range: cytochrome C, aprotinin, bacitracin, glycine–glycine–tyrosine–arginine, and glycine–glycine–glycine. The logarithm of molecular weight was plotted against retention time to establish the standard curve. The molecular weight distribution of peptides in the samples was quantitatively evaluated based on the relative peak areas using the area normalization method.

### 2.8. Flavor Analysis

Volatile flavor compounds were analyzed using a Headspace-Solid Phase Microextraction Gas Chromatography-Mass Spectrometry system (HS-SPME-GC-MS, GC-MS-QP2020 NX, Shimadzu Corporation, Kyoto, Japan) following a method from Zhang Q. et al. [33]. A 10 mL aliquot of broth was transferred to a 20 mL headspace vial, followed by the addition of 1 g sodium chloride to enhance volatility, along with 1 μL of internal standard solution (118.2 ppm 2-methyl-3-heptanone in methanol). The vial was sealed with a polytetrafluoroethylene (PTFE) septum and subjected to headspace adsorption at 50 °C for 31 min. The adsorbed volatiles were then thermally desorbed in the GC injection port at 250 °C for 7 min.

Chromatographic separation was performed using an SH-Wax capillary column (30 m × 0.25 mm, 0.25 μm film thickness). The injector temperature was maintained at 250 °C in splitless mode. The oven temperature program was as follows: initial temperature of 40 °C held for 3 min, increased to 100 °C at 5 °C/min, then raised to 230 °C at 10 °C/min, and finally held at 230 °C for 5 min. High-purity helium was employed as the carrier gas at a constant flow rate of 0.8 mL/min.

Mass spectrometric detection was conducted in electron ionization (EI) mode at 70 eV. The mass scanning range was set from 33 to 400 *m*/*z*. The ion source and interface temperatures were maintained at 200 °C and 250 °C, respectively. A solvent delay of 3 min was applied to protect the detector. Quantification of volatile compounds was performed using the internal standard method according to Equation (2):

(2) $$\mathrm{Concentration}\;(\mathrm{mg}/L)\;=\;\frac{A_{1}\;\times \;C}{V\;\times {\;A}_{2}}$$

where A_1_ represents the peak area of the target compound, C denotes the concentration of the internal standard (mg/L), V indicates the volume of broth sample (L), and A_2_ refers to the peak area of the internal standard.

### 2.9. Microstructure and Stability of Fat Globules

The microstructure of fat globules in the broth was examined using optical microscopy following a method from Su et al. [14]. Samples were maintained at 60 °C in a thermally insulated container to prevent fat crystallization prior to analysis. An aliquot of each warmed sample was placed on a preheated glass slide and immediately observed under a microscope (DS-Ri2, Nikon, Tokyo, Japan) equipped with a 20× objective lens. Images were captured to assess the size distribution and physical state of the fat globules.

The stability of the fat emulsion was evaluated using a dispersion analyzer (LUM GmbH, Berlin, Germany). The method is based on Zhou et al. [37], with minor modifications. Measurements were performed at 50 °C to simulate serving conditions. The samples were centrifuged at 3000 rpm through 150 rotational steps, with readings taken at 10-s intervals and a light transmission factor set at 1.0. Changes in transmission at 865 nm were monitored over a 25-min period to quantify the kinetics of phase separation and emulsion destabilization.

### 2.10. Antioxidant Capacity

#### 2.10.1. ABTS Radical Scavenging Activity

The ABTS radical cation solution was prepared by reacting 7 mmol/L ABTS with 2.45 mmol/L potassium persulfate in ultrapure water. The mixture was incubated in the dark at 25 °C for 12–16 h to generate ABTS^+^ radicals. Prior to analysis, the solution was diluted with 0.2 mol/L phosphate buffer (pH 7.4) until an absorbance of 0.700 ± 0.020 was achieved at 734 nm. A standard curve was constructed using Trolox solutions at concentrations ranging from 0 to 1000 μmol/L. For the assay, 10 μL of each standard or sample was mixed with 190 μL of the diluted ABTS^+^ solution in a 96-well microplate. After incubation in the dark at room temperature for 10 min, the absorbance was measured at 734 nm using a microplate reader, with phosphate-buffered saline serving as the blank control. The ABTS radical scavenging activity of the samples was quantified in μmol/g of sample, following the method described by Yu et al. [38].

#### 2.10.2. Ferric Ion-Reducing Antioxidant Power (FRAP)

The FRAP working solution was freshly prepared by mixing 10 mmol/L TPTZ (dissolved in 40 mmol/L HCl), 20 mmol/L ferric chloride, and 0.3 mol/L acetate buffer (pH 3.6) in a volume ratio of 1:1:10. The mixture was incubated at 37 °C for 1 h to form the Fe^3+^-TPTZ complex. In a 96-well plate, 10 μL of standard or sample was combined with 190 μL of the FRAP working solution and incubated at room temperature for 30 min in the dark. The absorbance was measured at 593 nm, with ultrapure water used as a blank. A standard curve was generated using Trolox solutions (0–1000 μmol/L), and the reducing power of the samples was expressed as μmol Trolox equivalents per gram (μmol TE/g) of sample, according to the procedure reported by Fan et al. [39].

### 2.11. Statistical Analysis

All experiments were conducted with three biologically independent replicates. Analysis of variance (ANOVA) and Duncan test were performed using SPSS 25.0 software (SPSS, Inc., Chicago, IL, USA). Graphs were prepared using Origin 2021 (version 9.8.0.200). *p* < 0.05 was considered statistically significant. Results were presented as mean ± standard deviation (SD).

## 3. Results and Discussion

### 3.1. Sensory Evaluation

**Figure 2:**
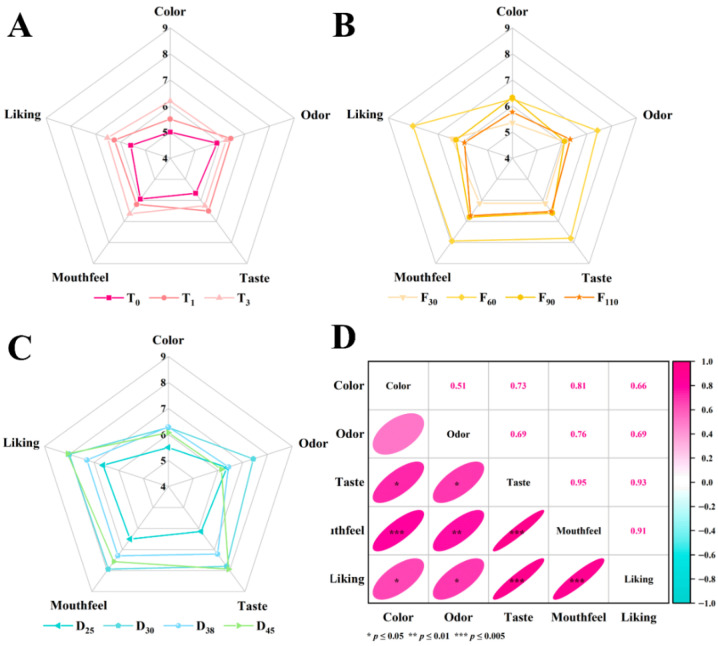
Radar chart ((**A**): ventilation time, (**B**): ventilation frequency, and (**C**): cooking duration) and correlation analysis (**D**) of pork keel bone broth under different controlled atmosphere conditions.

The sensory evaluation results of pork bone broth prepared under different CA conditions are summarized in Figure 2. CA treatment significantly improved the overall sensory scores, including color, odor, taste, mouthfeel, and overall liking, compared with the non-CA treated sample (T0). Under CA treatment, as ventilation time was increased from 0 s to 3 s, the total sensory score of pork bone broth exhibited an upward trend, rising from 28.06 to 31.84. These results indicate that appropriate extension of ventilation time can further optimize sensory quality of pork bone broth. With ventilation frequency increased, the overall sensory score of pork bone broth followed a pattern of initial increase followed by decline, the total sensory score peaked at 37.39 in the F60 sample. Subsequently, the overall sensory score gradually decreased as ventilation frequency was further elevated. This indicates that ventilation frequency has an optimal level, and both excessively high or low frequencies are detrimental to sensory quality. Under CA conditions (3 s and 60 cycles of ventilation), extending the cooking time beyond 30 min resulted in a progressive decrease in color and odor scores. In contrast, taste and mouthfeel scores decreased initially but subsequently increased with prolonged cooking. And no significant differences (*p <* 0.05) were observed in the total sensory scores between cooking durations of 30 and 45 min. It was hypothesized that extended cooking promotes the dissolution of compounds, thereby enhancing mouthfeel [40,41]. However, prolonged heating also intensified oxidation and MR, which contributed to the deterioration of odor and taste. In addition, umami nucleotides such as inosine monophosphate (IMP) and guanosine monophosphate (GMP) may degrade over prolonged heating time, and some FAA could also break down, leading to further alterations in taste profile [42,43]. As a result, the sensory evaluation indicated that CA processing enhanced mechanical agitation, facilitating the release of soluble components and improving mouthfeel. Nevertheless, it also accelerated oxidation and MR, which exerted adverse effects on flavor attributes. Therefore, achieving a balance between maximizing extraction efficiency and preserving desirable sensory qualities is crucial for producing high-quality broth with superior taste within a shorter processing time. Effective control of oxidation and flavor degradation, while promoting beneficial characteristics, is essential for the efficient production of premium bone broth.

### 3.2. Basic Component Content

**Figure 3:**
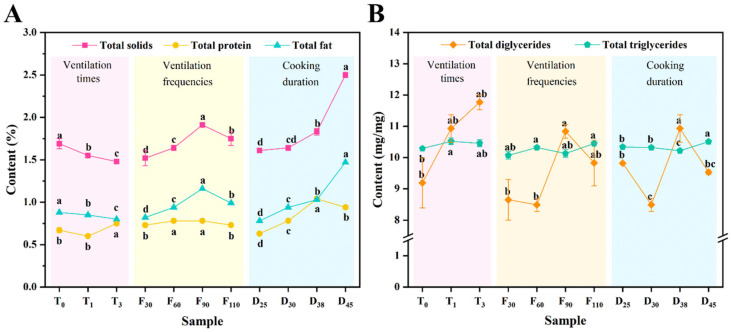
The total solid, protein, fat (**A**), diglycerides, and triglycerides (**B**) contents of pork keel bone broth under different controlled atmosphere conditions. Lowercase letters indicate statistically significant differences (*p* < 0.05).

The contents of total solids, protein, fat, DG, and TG in pork keel bone broth prepared under different CA conditions are summarized in Figure 3. As ventilation time increased from 0 to 3 s, both the total solid and fat contents decreased significantly (*p* < 0.05), from 1.69% to 1.48% and from 0.88% to 0.80%, respectively. Protein content initially decreased from 0.67% to 0.60%, before increasing to 0.75%. This pattern may be attributed to the agitation introduced by ventilation, which reduced the broth temperature, thereby slowing the release of solids and fats while promoting protein dissolution [44]. Notably, despite the overall reduction in fat content, the DG content exhibited a continuous increase with extended ventilation time, suggesting that CA conditions may facilitate the hydrolysis of TG into DG.

Integration of compositional data with sensory evaluation outcomes indicated that although extended ventilation time improved color, mouthfeel, and overall liking scores, it exerted an adverse effect on taste and odor. This implies that higher protein content may contribute to improved mouthfeel, and the elevated DG levels—likely due to their emulsifying properties [45]—could positively influence color and texture. Furthermore, study has demonstrated that an appropriate level of fat content exerts a positive effect on the emulsification and interfacial characteristics of bone soup, thus contributing to the improvement of its sensory quality [46]. These beneficial effects may account for the sustained high overall liking of the broth, despite the reduction in total solid content.

As ventilation frequency increased from 30 to 90 cycles, significant rises (*p* < 0.05) were observed in the contents of total solids, fat, and DG, with a modest increase in protein. The highest values were recorded in the F90 sample, reaching 1.91% for total solids, 1.16% for fat, 10.84% for DG, and 0.78% for protein. However, at 110 cycles, all component contents declined except for TG, implying that excessive ventilation may inhibit component extraction even under controlled temperature conditions. For instance, excessive oxygen exposure results in lipid oxidation [47]. Sensory evaluation results correlated with these trends, showing the highest total score at 60 cycles, beyond which further increases in cycle number resulted in reduced acceptability. The correlation between color attributes and DG levels underscores the importance of emulsification in producing the desirable milky white appearance preferred by consumers.

Prolonging the cooking duration from 25 to 45 min led to steady increases in total solids and fat content, with values increasing from 1.61% to 2.50% and from 0.78% to 1.47%, respectively. This is consistent with the findings by Guan Haining et al. [48], who reported that nutrient contents increased with increasing cooking time within a certain period. In contrast, protein content slightly decreased after 38 min, falling from 1.04% to 0.94%. Considerable fluctuations in DG and TG levels were also observed throughout the extended cooking process. When combined with sensory data, these results suggest that excessive ventilation may promote oxidative reactions and the MR, adversely impacting odor, taste, and mouthfeel. These negative sensory changes could not be offset by the increases in solid content, protein, fat, or improved emulsification, ultimately leading to a reduction in overall liking.

### 3.3. Fatty Acid Content

Table 2 summarizes the total fatty acid content of pork keel bone broth under various CA conditions. Palmitic and stearic acids were the dominant saturated fatty acids, while oleic and linoleic acids were the primary unsaturated fatty acids, maintaining a saturated-to-unsaturated fatty acid ratio of approximately 2:3. Notably, the broth also contained the rare odd-chain fatty acid heptadecenoic acid (C17:1), which is typically found in small amounts in ruminant fats.

The composition of fatty acids decreased as ventilation time increased. Notably, oleic acid, linoleic acid, stearic acid, and palmitic acid showed the most pronounced changes, while other fatty acids remained relatively stable. As highlighted in Section 3.2, the total fat content exhibited a slight decline with increasing ventilation time, likely due to the corresponding reduction in temperature [44]. These findings suggested that agitation significantly influenced the dissolution rates of various fatty acids. Moreover, it indicated that although ventilation might promote oxidation, the short cooking time effectively mitigated any significant effects on fatty acid composition.

As ventilation frequency increased from 30 to 110 cycles, the proportion of unsaturated fatty acids exhibited a slight decline, while the proportion of saturated fatty acids showed a marginal increase. However, all fatty acids displayed variations. A more detailed analysis, integrating fatty acid composition with total fat content, revealed that as ventilation frequency increased from 30 to 90 cycles, the fat content in the broth also rose. However, at 110 cycles, the fat content decreased, which was likely attributable to excessive ventilation lowering the broth temperature, thus inhibiting the overall fat dissolution.

As cooking time extended from 25 to 45 min, there was little change in the proportion of unsaturated and saturated fatty acids, although total fat content continued to rise. Excessive dissolution of unsaturated fatty acids often indicates the degradation of structural components such as collagen, which is typically associated with a weakening of the flavor profile [49]. High temperatures or prolonged heating times at lower temperatures negatively impacted flavor [36,50]. While ventilation affected the fat content and the dissolution of specific fatty acids, it altered the overall fatty acid composition. However, when ventilation significantly influenced the broth’s temperature, its impact on fat dissolution and fatty acid composition became more pronounced.

### 3.4. Amino Acid Composition

The increase in free amino acids, such as glutamic acid, lysine, and methionine, in broth was associated with a more pleasant flavor profile [51]. Additionally, some FAAs, such as beta-alanine, asparagine, tyrosine, threonine, methionine, cysteine, leucine, isoleucine, tryptophan, glutamic acid, histidine, lysine, and phenylalanine, may be associated with astringency and overheated flavors [42]. Table 3 and Table 4 present the FAA and TAA contents of pork keel bone broth under various CA conditions. Regarding the FAA results in Table 3, fluctuations in levels were observed as ventilation time increased from 0 to 3 s, with the T0 and T3 exhibiting the highest total FAA content of 0.47 mg/g broth. When ventilation frequency increased from 30 to 110 cycles, total FAA content initially rose, peaking at 0.53 mg/g in the D90 sample before subsequently declining. Similarly, prolonging the cooking duration from 25 to 45 min led to an increase in FAA content, which reached a maximum of 0.58 mg/g at 38 min, followed by a decrease. Notably, the variation in FAA content closely mirrored changes in sensory evaluations, with the loss of certain FAA indicating the development of overheated flavors due to MR. This was reflected in the overall acceptability, which showed a similar trend.

Regarding the TAA results in Table 4, as ventilation time increased from 0 to 3 s, the levels of various TAA fluctuated, with the T3 sample achieving the highest TAA content of 4.87 mg/g broth. In contrast, when ventilation frequency increased from 30 to 110 cycles, TAA content increased steadily, peaking at 6.92 mg/g in the D90 sample. Likewise, as the cooking duration was extended from 25 to 45 min, TAA content increased, reaching a maximum of 5.15 mg/g at 38 min before declining. Overall, the variations in TAA content were closely aligned with changes in total protein content and sensory evaluations of mouthfeel [36].

### 3.5. Peptide Molecular Weight Distribution

**Figure 4:**
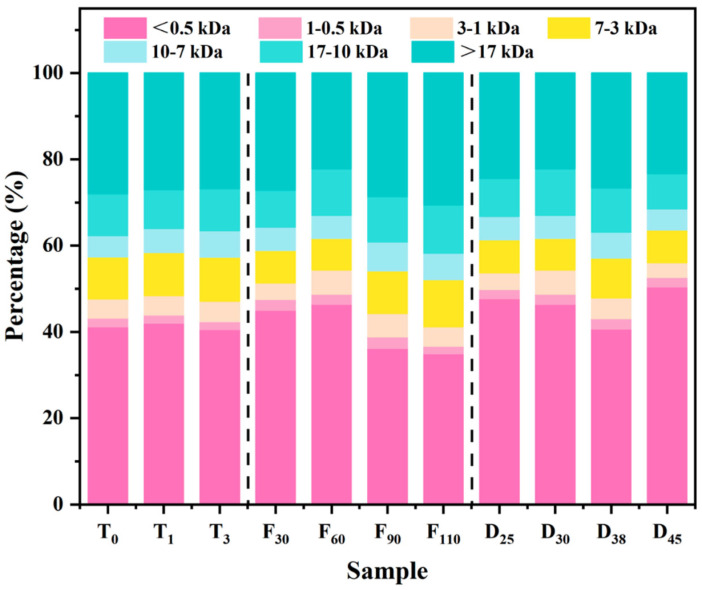
The peptide molecular weight distribution of pork keel bone broth under different controlled atmosphere conditions.

Figure 4 illustrates the peptide molecular weight distribution in pork keel bone broth under different CA conditions. As ventilation time was increased from 0 to 3 s, the proportion of peptides with molecular weights in the range of 1–7 kDa increased significantly. Sensory analysis further supported the conclusion that this shift positively influenced mouthfeel, mitigating the negative impact of decreased solid content due to lower temperature and thus maintaining high consumer preference. As ventilation frequency was increased from 30 to 110 cycles, the overall proportion of large peptides exhibited a decreasing trend. The relative abundances of most molecular weight fractions initially increased and then decreased, with the exception of peptides below 0.5 kDa, which displayed a continuous increase. This result, in conjunction with component data, suggested that excessive ventilation might reduce broth temperature and slightly diminish protein and fat solubility, while the increased agitation promoted further protein breakdown. These results were consistent with the previous observations of ventilation time. As the cooking duration extended from 25 to 45 min, the peptide molecular weight distribution became more complex. Peptides in the 17 kDa–3 kDa range increased, while peptides smaller than 3 kDa decreased after 30 min of cooking. This observation suggested that prolonged high-temperature cooking might intensify protein transformations, which corresponded with sensory and antioxidant results, where 30 min of cooking yielded the most favorable sensory results. Beyond this time, the broth’s odor and mouthfeel weakened, indicating that excessive MR negatively affected both flavor and health benefits. The distribution of dissolved protein molecular weights helped to explain the observed changes in flavor, mouthfeel, liking, and antioxidant activity [36]. Higher temperatures promoted collagen degradation and gelatin dissolution, leading to an increase in peptides within the 17 kDa-7 kDa and under 0.5 kDa ranges [52]. Peptides in the 7 kDa-1 kDa range, in particular, enhanced mouthfeel and flavor. Study has demonstrated that fish collagen-derived bioactive peptides can enhance the water-holding capacity and viscosity of yogurt [53], suggesting that collagen peptides possess thickening properties and thereby improve product texture. Ventilation-induced agitation accelerated protein degradation but also lowered broth temperature and increased the extent of oxidation and MR. The overall effect of ventilation depended on whether the positive impact of degradation outweighed the negative consequences of reduced temperature, enhanced oxidation, and MR.

### 3.6. Analysis of Flavor

The GC/MS analysis of pork bone broths prepared under different CA conditions is shown in Table 5. Following ventilation, the relative contents of multiple flavor compounds, including hexanal, heptanal, 2-hexenal, 2-pentylfuran, octanal, 2-heptenal, nonanal, 2-octenal, 1-octen-3-ol, benzaldehyde, 2-nonenal, octanol, and 2,4-decadienal, were elevated. As the ventilation time increased from 1 to 3 s, the contents of key flavor compounds, including heptanal, octanal, 2-hexenal, 2-pentylfuran, 2-octenal, 1-octen-3-ol, and 2-nonenal, showed a marked increase. However, alongside these flavor enhancements, the T3 sample also exhibited a greater accumulation of lipid oxidation products, such as heptanal and octanal, compared to the T1 sample. These results together with sensory evaluation, suggest that although the overall acceptability of the T3 sample was higher, the excessive accumulation of oxidation products did not necessarily enhance aroma, and the aroma differences between the T1 and T3 samples were negligible.

As ventilation frequency increased, the relative contents of key flavor compounds such as hexanal, heptanal, octanal, 2-heptenal, nonanal, 1-octen-3-ol, octanol, and 2,4-decadienal also rose, peaking at 90 cycles. Interestingly, 2-octenal showed a decreasing trend as ventilation frequency increased. Sensory evaluations indicated that the odor was most preferred at 60 cycles, aligning with the earlier observation that excessive accumulation of lipid oxidation products like heptanal and octanal might not necessarily improve aroma.

As ventilation time increased, the relative content of hexanal significantly increased, while the relative contents of heptanal, octanal, nonanal, 1-octen-3-ol, and octanol showed a slight increase. However, the changes during the 30 to 45-min ventilation duration were less pronounced. Sensory assessments indicated that the broth was most preferred at 30 min, suggesting that beyond this point, the continued accumulation of lipid oxidation products might negatively impact the aroma. The comparison between GC-MS and sensory evaluation results demonstrated a strong correlation, emphasizing that excessive accumulation of lipid oxidation products could negatively affect both aroma and overall sensory appeal [34].

### 3.7. Microstructure and Stability of Fat Globules

**Figure 5:**
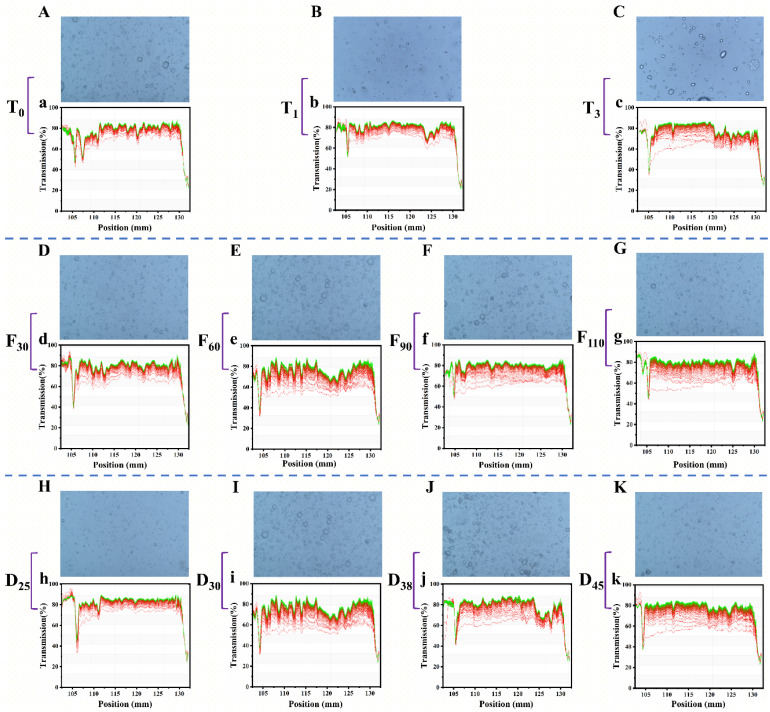
The images and Lumi results of pork keel bone broth under different controlled atmosphere conditions. (**A**, a) Microstructure and stability of fat globules in T_0_; (**B**, b) Microstructure and stability of fat globules in T_1_; (**C**, c) Microstructure and stability of fat globules in T_3_; (**D**, d) Microstructure and stability of fat globules in F_30_; (**E**, e) Microstructure and stability of fat globules in F_60_; (**F**, f) Microstructure and stability of fat globules in F_90_; (**G**, g) Microstructure and stability of fat globules in F_110_; (**H**, h) Microstructure and stability of fat globules in D_25_; (**I**, i) Microstructure and stability of fat globules in D_30_; (**J**, j) Microstructure and stability of fat globules in D_38_; (**K**, k) Microstructure and stability of fat globules in D_45_.

Fat derived from meat tissues is released into the broth and emulsified to form micro- and nano-scale oil droplets [40]. Images and Lumi results of pork bone broth prepared under different CA conditions are shown in Figure 5. As ventilation time was increased from 0 to 3 s, the microstructure shifted from a distribution of small droplets to a more uniform arrangement of larger droplets. The Lumi data indicated that the final profile curves of the T0 and T1 samples showed minimal changes from their initial states, suggesting clear stratification from the outset. In contrast, the T3 sample exhibited an initial transmittance of approximately 60%, indicative of superior emulsification properties under this condition. For samples with varying ventilation frequencies, the microstructure of the sample subjected to 30 ventilation cycles exhibited smaller and more evenly distributed droplets. The Lumi results also showed that as the ventilation frequency increased to 60 cycles or more, the initial transmittance of the bone broth decreased, indicating that higher ventilation frequencies enhanced stability [54]. In samples with different cooking times, the broth cooked for 25 min had smaller and more evenly distributed droplets. With prolonged cooking time, droplet density increased, indicative of enhanced fat release and dissolution. Lumi’s results further demonstrated that extending the cooking time reduced the initial transmittance from approximately 80% to 50%, suggesting that longer cooking times improved the emulsification properties of the broth.

### 3.8. Antioxidant Capacity

**Figure 6:**
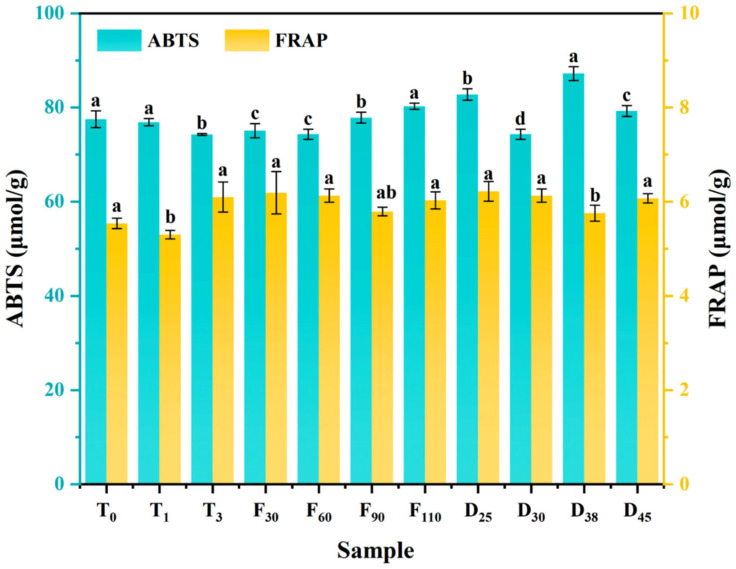
The ABTS radical scavenging activity and ferric ion-reducing antioxidant power of pork keel bone broth under different controlled atmosphere conditions. Lowercase letters indicate statistically significant differences (*p* < 0.05).

Figure 6 presents the FRAP and ABTS radical scavenging activities of pork keel bone broth under CA conditions. As ventilation time increased from 0 to 3 s, ABTS radical scavenging activity consistently decreased, with the T0 sample showing the highest value at 77.50 μmol/g. In contrast, FRAP values initially decreased significantly (*p* < 0.05) from 5.54 μmol/g to 5.30 μmol/g, before increasing significantly (*p* < 0.05) to 6.10 μmol/g. Increasing ventilation frequency from 30 to 110 cycles had no significant effect on FRAP values (*p* > 0.05), although ABTS activity rose steadily, peaking at 80.27 μmol/g in the F110 sample. Extending the cooking duration from 25 to 45 min had little impact on FRAP values, but ABTS activity followed a complex trend, reaching a maximum of 87.22 μmol/g at 38 min before declining. These findings suggested that ABTS radical scavenging capacity was influenced not only by dissolved component levels but also by the MR. While both oxidation and MR negatively influenced the broth’s flavor, moderate MR enhanced antioxidant properties. Thus, managing reactions such as MR was essential for optimizing both the flavor and functional properties of pork broth under CA conditions.

## 4. Conclusions

This study demonstrated that ventilation time, frequency, and cooking duration collectively modulate the sensory properties, nutritional profile, and flavor composition of pork bone broth. Relative to untreated control samples, appropriate adjustment of aeration conditions—including prolonged aeration time (3 s), elevated aeration frequency (60 cycles/min), and optimal cooking duration (30 min)—facilitated protein solubilization and flavor compound generation in pork bone broth. These optimal conditions elevated the content of low-molecular-weight peptides, improved overall sensory quality, and strengthened the emulsifying and antioxidant capacities of the broth. This study has certain limitations: dissolved oxygen levels were not accurately quantified, and only individual effects of CA parameters were investigated instead of multifactorial optimization. Future work will adopt multifactorial optimization designs to elucidate the interactive mechanisms of CA parameters, construct predictive quality control models, and lay a comprehensive theoretical foundation for the precise industrial application of CA technology in bone broth production.

## Figures and Tables

**Table 1:** Controlled atmosphere conditions for pork bone broths.

Parameter	T_0_	T_1_	T_3_	F_30_	F_60_	F_90_	F_110_	D_25_	D_30_	D_38_	D_45_
Ventilation time (s)	0	1	3	1	1	1	1	1	1	1	1
Ventilation frequency (cycle)	/	/	/	30	60	90	110	60	60	60	60
Cooking temperature (°C)	110	105	105	110	110	110	110	110	110	110	110
Cooking duration (min)	30	30	30	30	30	30	30	25	30	38	45

Note: F60 = D30.

**Table 2:** The total fatty acid content of pork keel bone broth under different controlled atmosphere conditions.

Category	T_0_	T_1_	T_3_	F_30_	F_60_	F_90_	F_110_	D_25_	D_30_	D_38_	D_45_	*p*
C10:0	0.06 ± 0.00 ^a^	0.05 ± 0.00 ^cd^	0.04 ± 0.00 ^f^	0.06 ± 0.00 ^b^	0.05 ± 0.00 ^d^	0.06 ± 0.00 ^c^	0.06 ± 0.00 ^cd^	0.06 ± 0.00 ^a^	0.05 ± 0.00 ^d^	0.05 ± 0.00 ^e^	0.06 ± 0.00 ^a^	<0.001
C11:0	10.00 ± 0.00	10.00 ± 0.00	10.00 ± 0.00	10.00 ± 0.00	10.00 ± 0.00	10.00 ± 0.00	10.00 ± 0.00	10.00 ± 0.00	10.00 ± 0.00	10.00 ± 0.00	10.00 ± 0.00	0.508
C12:0	0.07 ± 0.00 ^a^	0.05 ± 0.00 ^c^	0.05 ± 0.00 ^c^	0.06 ± 0.00 ^b^	0.06 ± 0.00 ^b^	0.05 ± 0.00 ^c^	0.06 ± 0.01 ^b^	0.07 ± 0.00 ^a^	0.06 ± 0.00 ^b^	0.05 ± 0.00 ^c^	0.06 ± 0.00 ^b^	<0.001
C14:0	1.00 ± 0.01 ^a^	0.77 ± 0.01 ^c^	0.58 ± 0.01 ^e^	0.84 ± 0.05 ^bc^	0.78 ± 0.02 ^c^	0.92 ± 0.03 ^a^	0.88 ± 0.00 ^b^	0.89 ± 0.01 ^b^	0.78 ± 0.02 ^c^	0.72 ± 0.05 ^d^	0.86 ± 0.01 ^b^	<0.001
C15:0	0.03 ± 0.00 ^a^	0.02 ± 0.00 ^c^	0.01 ± 0.00 ^d^	0.02 ± 0.00 ^b^	0.02 ± 0.00 ^b^	0.02 ± 0.01 ^b^	0.02 ± 0.00 ^b^	0.03 ± 0.00 ^a^	0.02 ± 0.00 ^b^	0.03 ± 0.00 ^a^	0.03 ± 0.00 ^a^	0.001
C16:0	19.65 ± 0.11 ^a^	16.27 ± 0.42 ^c^	12.50 ± 0.22 ^f^	17.40 ± 1.11 ^c^	15.81 ± 0.23 ^d^	19.76 ± 1.08 ^a^	18.61 ± 0.57 ^b^	18.31 ± 0.24 ^b^	15.81 ± 0.23 ^d^	14.93 ± 1.06 ^e^	18.67 ± 0.32 ^b^	<0.001
C16:1	2.5 ± 0.00 ^a^	1.56 ± 0.03 ^de^	1.52 ± 0.02 ^e^	2.01 ± 0.12 ^a^	1.78 ± 0.03 ^c^	2.04 ± 0.06 ^a^	2.08 ± 0.00 ^a^	1.90 ± 0.01 ^b^	1.78 ± 0.03 ^c^	1.44 ± 0.14 ^f^	1.62 ± 0.02 ^d^	<0.001
C17:0	0.19 ± 0.00 ^bc^	0.14 ± 0.00 ^f^	0.10 ± 0.00 ^g^	0.18 ± 0.01 ^c^	0.15 ± 0.00 ^e^	0.21 ± 0.010 ^b^	0.17 ± 0.00 ^cd^	0.20 ± 0.00 ^b^	0.15 ± 0.00 ^e^	0.16 ± 0.01 ^de^	0.27 ± 0.01 ^a^	<0.001
C17:1	0.17 ± 0.00 ^b^	0.12 ± 0.00 ^e^	0.10 ± 0.00 ^f^	0.16 ± 0.01 ^bc^	0.13 ± 0.00 ^d^	0.19 ± 0.01 ^a^	0.16 ± 0.00 ^bc^	0.15 ± 0.00 ^cd^	0.13 ± 0.00 ^d^	0.12 ± 0.01 ^e^	0.20 ± 0.00 ^a^	<0.001
C18:0	11.04 ± 0.03 ^a^	9.82 ± 0.22 ^c^	6.90 ± 0.11 ^e^	9.58 ± 0.62 ^c^	8.78 ± 0.14 ^d^	11.01 ± 0.54 ^a^	10.39 ± 0.23 ^b^	10.67 ± 0.25 ^b^	8.78 ± 0.14 ^d^	8.58 ± 0.62 ^d^	11.63 ± 0.23 ^a^	<0.001
C18:1,c+t	34.48 ± 0.16 ^a^	29.10 ± 0.73 ^cd^	22.31 ± 0.35 ^f^	30.64 ± 2.00 ^bc^	27.07 ± 0.37 ^d^	33.93 ± 1.89 ^a^	32.33 ± 0.96 ^b^	30.23 ± 0.47 ^c^	27.07 ± 0.37 ^d^	24.68 ± 1.82 ^e^	31.13 ± 0.57 ^b^	<0.001
C18:2,trans	8.00 ± 0.04 ^bc^	9.17 ± 0.16 ^a^	3.20 ± 0.02 ^f^	7.59 ± 0.45 ^cd^	6.66 ± 0.14 ^e^	8.25 ± 0.35 ^b^	6.59 ± 0.05 ^e^	9.41 ± 0.10 ^a^	6.66 ± 0.14 ^e^	7.36 ± 0.55 ^de^	7.86 ± 0.11 ^c^	<0.001
C18:3n3	0.33 ± 0.00 ^c^	0.48 ± 0.00 ^a^	0.10 ± 0.00 ^f^	0.310 ± 0.02 ^cd^	0.28 ± 0.01 ^de^	0.31 ± 0.01 ^cd^	0.23 ± 0.00 ^e^	0.43 ± 0.00 ^b^	0.28 ± 0.01 ^de^	0.26 ± 0.03 ^cd^	0.31 ± 0.00 ^cd^	<0.001
C20:0	0.13 ± 0.00 ^ab^	0.13 ± 0.00 ^ab^	0.08 ± 0.00 ^e^	0.12 ± 0.01 ^bc^	0.11 ± 0.00 ^cd^	0.13 ± 0.00 ^ab^	0.11 ± 0.00 ^cd^	0.13 ± 0.01 ^ab^	0.11 ± 0.00 ^cd^	0.13 ± 0.01 ^ab^	0.14 ± 0.00 ^a^	<0.001
C20:1	0.72 ± 0.00 ^ab^	0.62 ± 0.01 ^cd^	0.45 ± 0.00 ^e^	0.63 ± 0.05 ^cd^	0.58 ± 0.01 ^d^	0.71 ± 0.03 ^ab^	0.66 ± 0.00 ^bc^	0.75 ± 0.04 ^a^	0.58 ± 0.01 ^d^	0.75 ± 0.06 ^a^	0.72 ± 0.01 a^b^	<0.001
C20:2	0.48 ± 0.00 ^c^	0.54 ± 0.01 ^b^	0.21 ± 0.00 ^f^	0.44 ± 0.03 ^d^	0.41 ± 0.01 ^e^	0.50 ± 0.02 ^bc^	0.41 ± 0.00 ^e^	0.59 ± 0.01 ^a^	0.41 ± 0.01 ^e^	0.46 ± 0.03 ^cd^	0.49 ± 0.01 ^bc^	<0.001
C21:0	0.08 ± 0.00 ^b^	0.06 ± 0.00 ^d^	0.03 ± 0.00 ^e^	0.08 ± 0.00 ^b^	0.07 ± 0.00 ^c^	0.08 ± 0.00 ^b^	0.07 ± 0.00 ^c^	0.10 ± 0.00 ^a^	0.07 ± 0.00 ^c^	0.06 ± 0.01 ^d^	0.08 ± 0.00 ^b^	<0.001
C20:3n3	0.22 ± 0.00 ^b^	0.15 ± 0.00 ^e^	0.11 ± 0.00 ^f^	0.22 ± 0.01 ^b^	0.18 ± 0.00 ^d^	0.19 ± 0.01 ^cd^	0.20 ± 0.00 ^bc^	0.24 ± 0.01 ^a^	0.18 ± 0.00 ^d^	0.12 ± 0.01 ^f^	0.19 ± 0.00 ^cd^	<0.001
C20:4	0.07 ± 0.00 ^c^	0.10 ± 0.00 ^a^	0.02 ± 0.00 ^f^	0.05 ± 0.01 ^de^	0.06 ± 0.00 ^cd^	0.06 ± 0.00 ^cd^	0.05 ± 0.00 ^de^	0.09 ± 0.01 ^b^	0.06 ± 0.00 ^cd^	0.06 ± 0.01 ^cd^	0.06 ± 0.00 ^cd^	<0.001
C22:2	0.11 ± 0.03 ^ab^	0.06 ± 0.00 ^cd^	0.07 ± 0.01 ^bcd^	0.10 ± 0.00 ^abc^	0.08 ± 0.00 ^bcd^	0.11 ± 0.02 ^ab^	0.13 ± 0.04 ^a^	0.07 ± 0.02 ^bcd^	0.08 ± 0.00 ^bcd^	0.08 ± 0.01 ^bcd^	0.10 ± 0.01 ^abc^	0.012
Saturated fatty acid	42.25 ± 0.11 ^a^	37.31 ± 0.47 ^d^	30.29 ± 0.25 ^f^	38.34 ± 1.27 ^c^	35.83 ± 0.27 ^e^	42.24 ± 1.21 ^a^	40.37 ± 0.61 ^b^	40.46 ± 0.35 ^b^	35.83 ± 0.27 ^e^	34.71 ± 1.23 ^e^	41.80 ± 0.39 ^ab^	<0.001
Unsaturated fatty acid	47.08 ± 0.17 ^a^	41.90 ± 0.75 ^c^	28.09 ± 0.35 ^f^	42.15 ± 2.05 ^bc^	37.23 ± 0.40 ^d^	46.29 ± 1.92 ^a^	42.84 ± 0.96 ^b^	43.86 ± 0.48 ^b^	37.23 ± 0.40 ^d^	35.33 ± 1.91 ^d^	42.68 ± 0.58 ^bc^	<0.001

Lowercase letters indicate statistically significant differences.

**Table 3:** Free amino acid composition in pork keel bone broth under different controlled atmosphere conditions.

	Free Amino Acid Content (mg/g broth)											
Category	Ventilation Time			Ventilation Frequency				Cooking Duration				*p*
	T0	T1	T3	F30	F60	F90	F110	D25	D30	D38	D45	
Aspartic acid	0.01 ± 0.00 ^c^	0.01 ± 0.00 ^ef^	0.01 ± 0.00 ^cd^	0.01 ± 0.00 ^def^	0.01 ± 0.00 ^cde^	0.02 ± 0.00	0.01 ± 0.00 ^ef^	0.01 ± 0.00 ^c^	0.01 ± 0.00 ^cde^	0.02 ± 0.00 ^a^	0.01 ± 0.00 ^f^	<0.001
Glutamic acid	0.05 ± 0.01 ^cd^	0.05 ± 0.00 ^d^	0.06 ± 0.00 ^b^	0.05 ± 0.00 ^cd^	0.06 ± 0.00 ^bc^	0.06 ± 0.00 ^b^	0.05 ± 0.00 ^cd^	0.05 ± 0.00 ^cd^	0.06 ± 0.00 ^bc^	0.08 ± 0.00 ^a^	0.05 ± 0.00 ^cd^	<0.001
Serine	0.00 ± 0.00 ^c^	0.00 ± 0.00 ^ab^	0.00 ± 0.00 ^a^	0.00 ± 0.00 ^a^	0.00 ± 0.00 ^c^	0.00 ± 0.00 ^c^	0.00 ± 0.00 ^c^	0.00 ± 0.00 ^c^	0.00 ± 0.00 ^c^	0.00 ± 0.00 ^bc^	0.00 ± 0.00 ^c^	0.015
Histidine	0.01 ± 0.00	0.01 ± 0.00	0.01 ± 0.00	0.01 ± 0.00	0.01 ± 0.00	0.01 ± 0.00	0.01 ± 0.00	0.05 ± 0.00	0.01 ± 0.00	0.01 ± 0.00	0.01 ± 0.00	0.003
Glycine	0.02 ± 0.02	0.02 ± 0.00	0.03 ± 0.00	0.03 ± 0.01	0.03 ± 0.00	0.03 ± 0.00	0.03 ± 0.00	0.02 ± 0.00	0.03 ± 0.00	0.04 ± 0.00	0.02 ± 0.00	0.048
Threonine	0.01 ± 0.01	0.01 ± 0.00	0.02 ± 0.00	0.02 ± 0.01	0.02 ± 0.00	0.02 ± 0.00	0.01 ± 0.00	0.01 ± 0.00	0.02 ± 0.00	0.02 ± 0.00	0.01 ± 0.01	0.525
Arginine	0.03 ± 0.00 ^ab^	0.03 ± 0.00 ^d^	0.01 ± 0.00 ^d^	0.01 ± 0.00 ^d^	0.03 ± 0.00 ^bc^	0.03 ± 0.00 ^a^	0.03 ± 0.00 ^ab^	0.17 ± 0.00 ^ab^	0.03 ± 0.00 ^bc^	0.03 ± 0.01 ^a^	0.03 ± 0.00 ^c^	<0.001
Alanine	0.20 ± 0.03 ^abc^	0.20 ± 0.01 ^abc^	0.19 ± 0.00 ^bc^	0.22 ± 0.02 ^a^	0.21 ± 0.00 ^ab^	0.19 ± 0.00 ^bc^	0.22 ± 0.01 ^a^	0.1 ± 0.00 ^d^	0.21 ± 0.00 ^ab^	0.18 ± 0.01 ^cd^	0.22 ± 0.00 ^a^	0.002
Tyrosine	0.03 ± 0.01 ^cd^	0.03 ± 0.00 ^cd^	0.04 ± 0.00 ^a^	0.03 ± 0.00 ^abc^	0.03 ± 0.00 ^bcd^	0.03 ± 0.00 ^bcd^	0.03 ± 0.00 ^bd^	0.02 ± 0.00 ^bd^	0.03 ± 0.00 ^bcd^	0.03 ± 0.00 ^ab^	0.03 ± 0.00 ^d^	0.016
Cysteine	0.00 ± 0.00 ^ab^	0.00 ± 0.00 ^abc^	0.00 ± 0.00 ^abc^	0.00 ± 0.00 ^c^	0.00 ± 0.00 ^a^	0.00 ± 0.00 ^bc^	0.00 ± 0.00 ^bc^	0.00 ± 0.00 ^ab^	0.00 ± 0.00 ^a^	0.00 ± 0.00 ^abc^	0.00 ± 0.00 ^c^	0.013
Valine	0.02 ± 0.00 ^bc^	0.02 ± 0.00 ^e^	0.02 ± 0.00 ^de^	0.02 ± 0.00 ^cd^	0.02 ± 0.00 ^c^	0.03 ± 0.00 ^b^	0.02 ± 0.00 ^cde^	0.02 ± 0.00 ^bcd^	0.02 ± 0.00 ^c^	0.03 ± 0.00 ^a^	0.02 ± 0.00 ^c^	<0.001
Methionine	0.01 ± 0.00 ^bc^	0.01 ± 0.00 ^bc^	0.01 ± 0.00 ^ab^	0.01 ± 0.00 ^a^	0.01 ± 0.00 ^abc^	0.01 ± 0.00 ^a^	0.01 ± 0.00 ^bc^	0.01 ± 0.00 ^c^	0.01 ± 0.00 ^abc^	0.01 ± 0.00 ^a^	0.01 ± 0.00 ^ab^	0.013
Phenylalanine	0.01 ± 0.00 ^cd^	0.01 ± 0.00 ^e^	0.01 ± 0.00 ^cde^	0.01 ± 0.00 ^cde^	0.02 ± 0.00 ^bc^	0.02 ± 0.00 ^b^	0.01 ± 0.00 ^de^	0.02 ± 0.00 ^bc^	0.02 ± 0.00 ^bc^	0.02 ± 0.00 ^a^	0.02 ± 0.00 ^bcd^	<0.001
Isoleucine	0.01 ± 0.00 ^cd^	0.01 ± 0.00 ^d^	0.01 ± 0.00 ^c^	0.01 ± 0.00 ^cd^	0.01 ± 0.00 ^cd^	0.01 ± 0.00 ^b^	0.01 ± 0.00 ^cd^	0.01 ± 0.00 ^cd^	0.01 ± 0.00 ^cd^	0.02 ± 0.00 ^a^	0.01 ± 0.00 ^cd^	<0.001
Leucine	0.02 ± 0.00 ^cd^	0.02 ± 0.00 ^cd^	0.02 ± 0.00 ^bc^	0.02 ± 0.00 ^bcd^	0.02 ± 0.00 ^bc^	0.03 ± 0.00 ^b^	0.01 ± 0.00 ^d^	0.02 ± 0.00 ^bc^	0.02 ± 0.00 ^bc^	0.04 ± 0.00 ^a^	0.02 ± 0.00 ^bc^	<0.001
Lysine	0.02 ± 0.00 ^cd^	0.02 ± 0.00 ^f^	0.01 ± 0.00 ^def^	0.01 ± 0.00 ^ef^	0.02 ± 0.00 ^cd^	0.02 ± 0.00 ^b^	0.01 ± 0.00 ^f^	0.02 ± 0.00 ^c^	0.02 ± 0.00 ^cd^	0.02 ± 0.00 ^a^	0.02 ± 0.00 ^cde^	<0.001
Proline	0.01 ± 0.00 ^cd^	0.01 ± 0.00 ^bcd^	0.02 ± 0.00 ^bc^	0.02 ± 0.00 ^b^	0.01 ± 0.00 ^bcd^	0.02 ± 0.00 ^b^	0.01 ± 0.00 ^d^	0.01 ± 0.00 ^bcd^	0.01 ± 0.00 ^bcd^	0.02 ± 0.00 ^a^	0.01 ± 0.00 ^cd^	0.003
Total	0.47 ± 0.10 ^bc^	0.44 ± 0.02 ^c^	0.47 ± 0.00 ^bc^	0.49 ± 0.01 ^bc^	0.51 ± 0.02 ^abc^	0.53 ± 0.01 ^ab^	0.48 ± 0.03 ^bc^	0.45 ± 0.00 ^bc^	0.51 ± 0.02 ^abc^	0.58 ± 0.02 ^a^	0.48 ± 0.04 ^bc^	0.003

Lowercase letters indicate statistically significant differences.

**Table 4:** Total amino acid composition in pork keel bone broth under different controlled atmosphere conditions.

	Total Amino Acid Content (mg/g broth)											
Category	Ventilation Time			Ventilation Frequency				Cooking Duration				*p*
	T0	T1	T3	F30	F60	F90	F110	D25	D30	D38	D45	
Aspartic acid	0.31 ± 0.05 ^d^	0.27 ± 0.01 ^d^	0.35 ± 0.02 ^cd^	0.28 ± 0.02 ^d^	0.34 ± 0.05 ^cd^	0.54 ± 0.02 ^a^	0.49 ± 0.00 ^ab^	0.30 ± 0.04 ^d^	0.34 ± 0.05 ^cd^	0.42 ± 0.05 ^bc^	0.29 ± 0.05 ^d^	<0.001
Glutamic acid	0.58 ± 0.07 ^d^	0.58 ± 0.02 ^d^	0.72 ± 0.04 ^c^	0.54 ± 0.04 ^d^	0.64 ± 0.05 ^cd^	1.00 ± 0.04 ^a^	0.88 ± 0.01 ^b^	0.60 ± 0.07 ^d^	0.64 ± 0.05 ^cd^	0.75 ± 0.05 ^c^	0.58 ± 0.08 ^d^	<0.001
Serine	0.12 ± 0.01 ^d^	0.13 ± 0.01 ^d^	0.17 ± 0.01 ^c^	0.11 ± 0.01 ^d^	0.13 ± 0.01 ^d^	0.22 ± 0.01 ^a^	0.19 ± 0.00 ^b^	0.12 ± 0.01 ^d^	0.13 ± 0.01 ^d^	0.16 ± 0.00 ^c^	0.12 ± 0.02 ^d^	<0.001
Histidine	0.28 ± 0.06 ^cd^	0.34 ± 0.01 ^abc^	0.37 ± 0.02 ^ab^	0.26 ± 0.01 ^d^	0.34 ± 0.02 ^abc^	0.30 ± 0.00 ^bcd^	0.37 ± 0.01 ^a^	0.38 ± 0.05 ^a^	0.34 ± 0.02 ^abc^	0.30 ± 0.00 ^cd^	0.33 ± 0.02 ^abc^	0.008
Glycine	0.62 ± 0.04 ^de^	0.67 ± 0.01 ^cd^	0.91 ± 0.04 ^b^	0.53 ± 0.05 ^e^	0.59 ± 0.06 ^de^	1.09 ± 0.08 ^a^	1.04 ± 0.01 ^a^	0.61 ± 0.06 ^de^	0.59 ± 0.06 ^de^	0.77 ± 0.03 ^c^	0.55 ± 0.08 ^de^	<0.001
Threonine	0.10 ± 0.01 ^d^	0.10 ± 0.00 ^d^	0.13 ± 0.01 ^b^	0.10 ± 0.01 ^d^	0.11 ± 0.01 ^cd^	0.18 ± 0.00 ^a^	0.14 ± 0.00 ^b^	0.10 ± 0.01 ^d^	0.11 ± 0.01 ^cd^	0.13 ± 0.00 ^bc^	0.10 ± 0.01 ^d^	<0.001
Arginine	0.60 ± 0.08 ^de^	0.27 ± 0.01 ^f^	0.36 ± 0.02 ^f^	0.55 ± 0.04 ^e^	0.67 ± 0.05 ^cd^	0.84 ± 0.03 ^ab^	0.90 ± 0.01 ^a^	0.74 ± 0.03 ^bc^	0.67 ± 0.05 ^cd^	0.71 ± 0.04 ^cd^	0.66 ± 0.07 ^cd^	<0.001
Alanine	0.34 ± 0.03 ^c^	0.32 ± 0.01 ^c^	0.42 ± 0.02 ^b^	0.30 ± 0.03 ^c^	0.34 ± 0.03 ^c^	0.60 ± 0.04 ^a^	0.55 ± 0.00 ^a^	0.34 ± 0.05 ^c^	0.34 ± 0.03 ^c^	0.43 ± 0.03 ^b^	0.32 ± 0.06	<0.001
Tyrosine	0.05 ± 0.00 ^b^	0.07 ± 0.04 ^ab^	0.06 ± 0.00 ^b^	0.05 ± 0.00 ^b^	0.06 ± 0.00 ^b^	0.09 ± 0.00 ^a^	0.07 ± 0.00 ^ab^	0.05 ± 0.01 ^b^	0.06 ± 0.00 ^b^	0.07 ± 0.01 ^ab^	0.05 ± 0.01 ^b^	0.018
Cysteine	0.01 ± 0.00 ^b^	0.01 ± 0.00 ^ab^	0.01 ± 0.00 ^ab^	0.01 ± 0.00 ^ab^	0.01 ± 0.00 ^a^	0.01 ± 0.00 ^ab^	0.01 ± 0.00 ^ab^	0.01 ± 0.00 ^ab^	0.01 ± 0.00 ^a^	0.01 ± 0.00 ^ab^	0.01 ± 0.00 ^ab^	0.021
Valine	0.14 ± 0.01 ^c^	0.12 ± 0.00 ^c^	0.16 ± 0.02 ^bc^	0.13 ± 0.01 ^c^	0.15 ± 0.02 ^bc^	0.25 ± 0.01 ^a^	0.21 ± 0.00 ^b^	0.14 ± 0.02 ^c^	0.15 ± 0.02 ^bc^	0.18 ± 0.02 ^ab^	0.14 ± 0.03 ^c^	<0.001
Methionine	0.03 ± 0.00 ^c^	0.04 ± 0.00 ^c^	0.05 ± 0.00 ^ab^	0.03 ± 0.00 ^c^	0.04 ± 0.01 ^ab^	0.05 ± 0.01 ^ab^	0.06 ± 0.00 ^a^	0.03 ± 0.01 ^c^	0.04 ± 0.01 ^ab^	0.04 ± 0.00 ^c^	0.03 ± 0.00 ^c^	0.009
Phenylalanine	0.10 ± 0.01 ^cd^	0.09 ± 0.00 ^d^	0.12 ± 0.01 ^bc^	0.09 ± 0.01 ^d^	0.10 ± 0.01 ^cd^	0.17 ± 0.01 ^a^	0.14 ± 0.00 ^b^	0.10 ± 0.02 ^cd^	0.10 ± 0.01 ^cd^	0.13 ± 0.02 ^b^	0.09 ± 0.02 ^cd^	<0.001
Isoleucine	0.08 ± 0.01 ^de^	0.07 ± 0.00 ^e^	0.09 ± 0.01 ^cd^	0.07 ± 0.00 ^de^	0.09 ± 0.01 ^cd^	0.14 ± 0.01 ^a^	0.12 ± 0.00 ^b^	0.08 ± 0.01 ^de^	0.09 ± 0.01 ^cd^	0.10 ± 0.01 ^bc^	0.08 ± 0.01 ^de^	<0.001
Leucine	0.17 ± 0.02 ^e^	0.16 ± 0.00 ^e^	0.21 ± 0.02 ^bcd^	0.16 ± 0.01 ^e^	0.19 ± 0.02 ^cde^	0.31 ± 0.01 ^a^	0.25 ± 0.00 ^b^	0.17 ± 0.03 ^de^	0.19 ± 0.02 ^cde^	0.23 ± 0.02 ^bc^	0.17 ± 0.03 ^de^	<0.001
Lysine	0.20 ± 0.02 ^de^	0.18 ± 0.00 ^e^	0.24 ± 0.01 ^cd^	0.19 ± 0.01 ^de^	0.23 ± 0.02 ^cde^	0.36 ± 0.01 ^a^	0.29 ± 0.01 ^b^	0.21 ± 0.03 ^de^	0.23 ± 0.02 ^cde^	0.27 ± 0.02 ^bc^	0.20 ± 0.03 ^de^	<0.001
Proline	0.37 ± 0.02 ^d^	0.38 ± 0.04 ^d^	0.51 ± 0.05 ^c^	0.31 ± 0.02 ^d^	0.34 ± 0.02 ^d^	0.74 ± 0.00 ^a^	0.60 ± 0.02 ^b^	0.35 ± 0.04 ^d^	0.34 ± 0.02 ^d^	0.46 ± 0.03 ^c^	0.30 ± 0.05 ^d^	<0.001
Total	4.09 ± 0.47 ^cd^	3.79 ± 0.09 ^d^	4.87 ± 0.31 ^bc^	3.71 ± 0.27 ^d^	4.37 ± 0.40 ^bcd^	6.92 ± 0.28 ^a^	6.32 ± 0.08 ^a^	4.34 ± 0.34 ^cd^	4.37 ± 0.40 ^bcd^	5.15 ± 0.32 ^b^	4.03 ± 0.61 ^cd^	<0.001

Lowercase letters indicate statistically significant differences.

**Table 5:** GC/MS analysis in pork keel bone broth under different controlled atmosphere conditions.

Compound (μg/L broth)	T0	T1	T3	F30	F60	F90	F110	D25	D30	D38	D45
Pentanal	6.8	5.1	9.0	7.0	9.7	10.3	6.9	15.7	9.7	11.3	13.0
5-(2-Methylpropyl)-nonane	0.0	0.0	0.0	0.0	0.0	0.0	0.0	87.4	0.0	0.0	0.0
Hexanal	28.2	63.2	65.5	41.0	78.8	82.4	49.9	38.5	78.8	81.1	127.2
Hexadecane	1.4	0.0	0.0	1.8	1.0	0.0	0.0	0.0	1.0	1.4	0.0
Heptanal	3.2	14.2	22.4	3.4	6.8	9.9	8.5	2.8	6.8	7.3	5.4
Dodecane	1.7	0.0	0.0	1.3	2.1	0.0	0.0	3.9	2.1	0.0	0.0
2(E)-Hexenal	0.0	0.0	2.9	0.0	0.0	0.0	0.0	0.0	0.0	0.0	0.0
2,5-Dimethyl-undecane	0.0	0.0	0.0	0.0	0.0	0.0	0.0	0.0	2.1	0.0	0.0
2-Hexenal	0.0	0.0	0.0	0.0	0.0	0.0	0.0	0.0	0.0	0.2	0.0
3-Methyl-1-butanol	0.7	0.0	0.0	1.5	2.3	1.4	2.5	2.5	2.3	0.6	0.0
2-Pentyl-furan	1.1	5.0	26.4	1.1	1.2	2.3	1.7	2.6	1.2	2.4	2.1
5-Methyl-tetradecane	0.7	0.0	0.0	2.3	0.0	0.0	0.0	5.2	0.0	0.0	1.9
4,6-Dimethyl-dodecane	1.3	0.3	0.0	3.8	2.2	1.5	0.0	8.3	2.2	2.1	2.5
1-Pentanol	1.8	1.6	4.2	1.8	3.4	3.2	3.0	2.9	3.4	3.2	2.9
Octanal	2.5	21.4	27.1	3.5	7.2	7.8	7.0	1.9	7.2	4.3	4.4
1-Octen-3-one	0.0	2.0	0.0	0.0	0.0	0.0	0.0	0.0	0.0	0.0	0.0
(E)-2-Heptenal	1.9	16.5	22.0	2.0	5.1	7.5	0.0	6.5	5.1	7.7	4.5
2,5-Octanedione	1.8	2.4	2.5	5.5	6.0	5.6	4.0	3.4	6.0	6.5	6.9
3-Methyl-4-methylene-hexane	0.0	0.5	0.0	0.0	0.0	0.0	0.0	0.0	0.0	0.0	0.0
6-Methyl-5-hepten-2-one	0.1	1.6	1.4	0.1	0.3	0.5	0.2	0.0	0.3	0.0	0.0
1-Hexanol	1.8	0.8	2.0	0.0	0.0	0.0	0.0	1.6	0.0	0.8	0.8
2-Nonanone	0.0	0.0	0.3	0.0	0.0	0.0	0.0	0.0	0.0	0.0	0.0
Nonanal	5.5	51.1	39.5	7.1	11.3	13.1	12.2	4.9	11.3	5.1	8.2
1-Ethyl-1-methyl-cyclopentane	0.0	0.7	0.9	0.0	0.0	0.0	0.0	0.0	0.0	0.0	0.0
3-Octen-2-one	0.0	0.6	1.0	0.0	0.0	0.0	0.0	0.0	0.0	0.0	0.0
3-Ethyl-2-methyl-1,3-hexadiene	0.3	1.4	5.6	0.3	1.6	1.8	1.2	0.0	1.6	1.7	0.0
(E)-2-Octenal	8.9	7.2	13.6	3.7	4.3	2.3	1.9	0.0	4.3	2.1	0.0
(E)-4-Nonenal	1.0	2.0	0.8	0.0	0.0	0.0	0.0	0.0	0.0	0.0	0.0
Acetic acid	0.0	0.5	0.0	0.0	0.0	0.0	0.0	0.0	0.0	0.0	0.4
1-Octen-3-ol	2.3	7.0	15.4	2.4	7.7	7.7	6.4	2.5	7.7	7.8	5.8
1-Heptanol	0.8	2.8	6.9	1.0	2.3	2.0	2.1	1.1	2.3	1.7	1.1
2,4-Dimethyl-cyclohexanol	0.0	0.0	0.3	0.0	0.0	0.0	0.0	0.0	0.0	0.2	0.0
(E,E)-2,4-Heptadienal	0.0	1.2	1.8	0.0	0.0	0.4	0.0	0.0	0.0	0.5	0.0
Decanal	0.0	1.7	1.9	0.0	0.0	0.0	0.0	0.0	0.0	0.0	0.0
1-Ethenyl-cyclohexanol	0.0	0.7	0.7	0.0	0.0	0.0	0.0	0.0	0.0	0.0	0.0
Trans-3-nonen-2-one	0.8	0.0	0.2	0.0	0.0	0.0	0.0	0.0	0.0	0.0	0.0
Benzaldehyde	0.8	1.1	2.7	1.3	1.0	1.0	0.4	0.9	1.0	1.1	1.3
(E)-2-nonenal	0.4	4.7	9.2	0.4	1.2	1.2	0.8	0.3	1.2	0.7	0.8
4-Ethylcyclohexanol	0.3	0.7	1.6	0.2	0.8	0.6	0.0	0.0	0.8	0.7	1.3
1-Octanol	0.9	5.1	9.1	1.1	2.4	2.3	2.5	0.7	2.4	1.1	1.3
(Z)-2-Octen-1-ol	0.7	1.7	4.3	0.6	1.2	0.9	0.7	0.6	1.2	0.9	0.0
Benzeneacetaldehyde	0.0	0.4	0.0	0.0	0.0	0.0	0.0	0.0	0.0	0.0	0.0
Neral	0.0	0.5	0.0	0.0	0.0	0.0	0.0	0.0	0.0	0.0	0.0
(E,E)-2,4-Nonadienal	0.0	1.0	0.0	0.0	0.0	0.0	0.0	0.0	0.0	0.0	0.0
2,4-Dimethyl-benzaldehyde	5.0	0.0	0.0	7.5	9.9	4.9	4.4	10.6	9.9	5.8	4.7
(E,E)-2,4-Decadienal	1.8	9.0	8.7	1.8	3.5	3.3	1.8	2.6	3.5	3.0	0.0
Hexanoic acid	0.2	0.6	0.0	0.2	0.6	0.4	0.2	0.2	0.6	0.3	1.1
Phenylethyl alcohol	0.2	2.4	2.1	0.5	0.9	0.5	0.5	0.4	0.9	0.2	0.0

## Data Availability

The original contributions presented in this study are included in the article. Further inquiries can be directed to the corresponding author.
